# Ethnogeographic and inter-individual variability of human ABC transporters

**DOI:** 10.1007/s00439-020-02150-6

**Published:** 2020-03-23

**Authors:** Qingyang Xiao, Yitian Zhou, Volker M. Lauschke

**Affiliations:** Section of Pharmacogenetics, Department of Physiology and Pharmacology, Karolinska Institutet, 17177 Stockholm, Sweden

## Abstract

ATP-binding cassette (ABC) transporters constitute a superfamily of 48 structurally similar membrane transporters that mediate the ATP-dependent cellular export of a plethora of endogenous and xenobiotic substances. Importantly, genetic variants in *ABC* genes that affect gene function have clinically important effects on drug disposition and can be predictors of the risk of adverse drug reactions and efficacy of chemotherapeutics, calcium channel blockers, and protease inhibitors. Furthermore, loss-of-function of ABC transporters is associated with a variety of congenital disorders. Despite their clinical importance, information about the frequencies and global distribution of functionally relevant *ABC* variants is limited and little is known about the overall genetic complexity of this important gene family. Here, we systematically mapped the genetic landscape of the entire human *ABC* superfamily using Next-Generation Sequencing data from 138,632 individuals across seven major populations. Overall, we identified 62,793 exonic variants, 98.5% of which were rare. By integrating five computational prediction algorithms with structural mapping approaches using experimentally determined crystal structures, we found that the functional *ABC* variability is extensive and highly population-specific. Every individual harbored between 9.3 and 13.9 deleterious *ABC* variants, 76% of which were found only in a single population. Carrier rates of pathogenic variants in *ABC* transporter genes associated with autosomal recessive congenital diseases, such as cystic fibrosis or pseudoxanthoma elasticum, closely mirrored the corresponding population-specific disease prevalence, thus providing a novel resource for rare disease epidemiology. Combined, we provide the most comprehensive, systematic, and consolidated overview of ethnogeographic ABC transporter variability with important implications for personalized medicine, clinical genetics, and precision public health.

**Electronic supplementary material:**

The online version of this article (10.1007/s00439-020-02150-6) contains supplementary material, which is available to authorized users.

## Introduction

ATP-binding cassette (ABC) transporters are a superfamily of membrane proteins that, in humans, comprise 48 genes. ABC transporters catalyse the translocation of a wide spectrum of endogenous substrates across biological membranes, including amino acids, sugars, nucleosides, vitamins, lipids, bile acids, leukotrienes, prostaglandins, uric acid, antioxidants, as well as a multitude of natural toxins (Liang et al. 2015). In addition, ABC transporters mediate the export of a plethora of drug substrates, including calcium channel blockers, HIV protease inhibitors, vinca alkaloids, topoisomerase inhibitors, methotrexate, anthracyclines, and taxanes, into the extracellular space and are thus key modulators of drug resistance, particularly in oncology (Robey et al. 2018). Hence, *ABC* transporters are of specific clinical and regulatory interest for their involvement in drug–drug interactions (König et al. 2013; Marquez and Van Bambeke 2011; Zhang et al. 2018).

Genetic variants in *ABC* transporters contribute to the inter-individual variability in the risk of adverse drug reactions and treatment efficacy, and are key modulators of drug resistance. Arguably, the most studied are polymorphisms in *ABCB1* (encoding MDR1, P-gp), which have been associated with methotrexate clearance (Kim et al. 2012a), response to antiretroviral protease inhibitors (Coelho et al. 2013), as well as with pharmacokinetics, response, and toxicity of imatinib (Dulucq et al. 2008; Ma et al. 2017). Similarly, variants in *ABCG2* (encoding BCRP) were reproducibly associated with exposure and response to statins (Bailey et al. 2010; Chasman et al. 2012; Hu et al. 2011) and allopurinol (Roberts et al. 2017; Wen et al. 2015). In addition to their pharmacogenetic importance, genetic variation in 21 ABC transporters can cause congenital diseases, the most common of which is cystic fibrosis (OMIM 219700) caused by variants in *ABCC7* (CFTR).

Importantly, while many studies have provided critical data about the clinical importance of *ABC* polymorphisms (Bosch et al. 2005; Fukushima-Uesaka et al. 2007; Honjo et al. 2002; Leschziner et al. 2006; Pramanik et al. 2014; Saito et al. 2002; Słomka et al. 2015), information about their population frequencies is limited and mostly derived from relatively small, heterogeneous cohorts. Furthermore, most studies only interrogated a few selected candidate variants and did not map the entire landscape of rare genetic variability that is characteristic for pharmacogenes (Bush et al. 2016; Fujikura et al. 2015; Gordon et al. 2014; Ingelman-Sundberg et al. 2018; Kozyra et al. 2017; Wright et al. 2018; Zhou and Lauschke 2018). Importantly, the increasing prevalence of Next-Generation Sequencing (NGS) projects on a population scale allows for the first time to systematically parse the inter-individual and inter-population variability in *ABC* transporter superfamily.

In the current study, we systematically parsed the inter-individual and inter-population variability in the *ABC* transporter superfamily by analyzing whole-exome and whole-genome sequencing (WES and WGS, respectively) data from 138,632 individuals across seven major human populations. Using this large data set, we provide frequencies of 51 *ABC* variants and haplotypes frequencies with demonstrated clinical relevance. In addition to these well-characterized variations, we identified 62,793 exonic variants, the vast majority of which were rare and have not been characterized. Computational analyses using five partly orthogonal algorithms predicted that 19,309 of these (31%) resulted in functional alterations of the respective transporter protein. To substantiate these estimates, we mapped the identified genetic variability onto experimentally determined or homology-modeled transporter structures and found multiple amino acid exchanges in residues important for substrate binding and transporter function. The present study constitutes the most comprehensive analysis of genetic variation in the *ABC* superfamily published to date and the identified genetic complexity might have important implications for the evaluation of drug transporter variability during drug development and the personalized prediction of drug disposition, response, and toxicity.

## Methods

### Data collection and definitions

Single-nucleotide variant (SNV) and indel frequency data across 48 human ABC transporters were collected from WES and WGS data from 138,632 individuals (12,020 Africans, 17,210 Latinos, 5076 Ashkenazi Jews, 9435 East Asians, 15,391 South Asians, 12,897 Finns, 63,369 non-Finnish Europeans, and 3234 from other ethnic groups) acquired from the Genome Aggregation Database (Lek et al. 2016). Variants with MAF < 1% or MAF < 0.1% were defined as rare and very rare, respectively. Copy-number variation (CNV) data were extracted from the Exome Aggregation Consortium database using genomic information from 59,451 individuals and analyzed as previously described (Santos et al. 2018). Linkage disequilibria were computed by leveraging linkage from the 1000 Genomes Project using LDLink (Machiela and Chanock 2015). The Online Mendelian Inheritance in Man (OMIM) database was used to identify *ABC* genes associated with Mendelian disease, as well as their mode of inheritance (Amberger et al. 2015). One-way ANOVA was used to compare the difference between variant number across *ABC* subfamilies.

### Variant effect predictions

To predict the functional consequences of missense variants, we used a panel of computational algorithms that analyze sequence conservation, as well as variant effects on physicochemical amino acid properties, solvent accessibility, and structural features. Specifically, we selected SIFT (Ng and Henikoff 2001), Polyphen2 (Adzhubei et al. 2010), MutationAssessor (Reva et al. 2011), VEST3 (Carter et al. 2013), and Eigen (Ionita-Laza et al. 2016), as they showed the best predictive performance in three independent benchmarking data sets (Li et al. 2018a). Variants were categorized as deleterious when the ≥ 50% of algorithms predicted effects on transporter function. In addition, all frameshifts, in-frame deletions or insertions, start-lost, stop-gained, or canonical splice site variants were regarded as putatively deleterious. For Mendelian disease analyses, ClinVar (Landrum et al. 2014) was used to remove benign variants from disease-associated *ABC* genes.

### Structural analysis

We analyzed the impact of genetic variation on ABC transporter structures for the entire *ABCA*, *ABCB,* and *ABCC* transporter families (35 proteins in total). Experimentally determined crystal structures were available for 18 ABC transporter proteins and were extracted from PDB (Berman et al. 2000) and the available literature. The remaining 16 transporter structures were modeled based on homology using Phyre2 (Kelley et al. 2015). The structure of ABCA13 could not be modeled reliably and was thus excluded. PyMOL (version 2.1.1) was used to map the genetic variability data onto the corresponding transporter structures.

## Results

### Genetic variability of the human *ABC* transporter superfamily

**Fig. 1:**
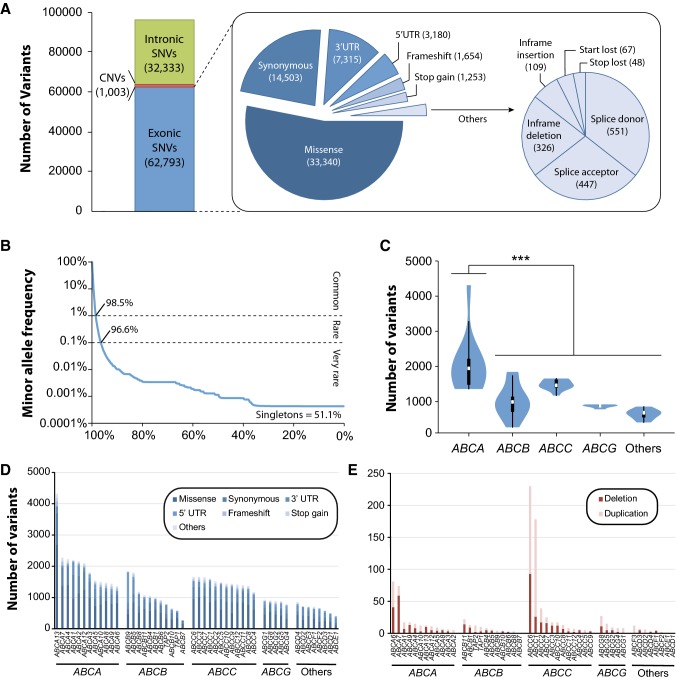
Overview of the genetic germline variability in the human ABC transporter family. **a** In total 62,793 exonic variants and 1003 copy-number variations (CNVs) were identified across all 48 human *ABC* genes in 138,632 individuals. **b** The vast majority of exonic *ABC* variants were rare with 98.5% occurring in less than 1% of alleles worldwide. In addition, 51.1% of all variants were only found in a single individual. **c***ABCA* genes harbour significantly more variations than members of other ABC subfamilies (*p* = 0.002; ANOVA). These differences were mostly related to gene length (compare Supplementary Figure 1). **d** Stacked column plot depicting the number of variants across variants classes for all 48 *ABC* genes. **e** The number of CNVs that affect at least one exon are shown

We systematically analyzed the genetic variability profiles of all 48 members of the human *ABC* transporter gene superfamily using NGS data from 138,632 individuals. In total, we identified 62,793 variants in exons, the majority of which were missense (*n* = 33,340; 53%), followed by synonymous (*n* = 14,503; 23%) and UTR variations (*n* = 10,495; 17%; Fig. 1a). Importantly, the vast majority of variations (*n* = 61,876; 98.5%) were rare with minor allele frequencies (MAF) < 1%, whereas only 917 (1.5%) variations were common (Fig. 1b). In addition, we found 1003 deletions or duplications spanning at least one *ABC* exon, jointly referred to as CNVs, as well as 32,333 intronic variants. The latter were, however, not systematically covered and thus excluded from further analyses.

Notably, the number of genetic variations differed considerably between *ABC* subfamilies and genes. Overall, the number of variants in the *ABCA* family of lipid transporters was significantly higher than in other *ABC* subfamilies (*p* = 0.002; fold difference = 1.9; Fig. 1c). Of all members of the human *ABC* superfamily of genes, the lipid transporters *ABCA13* (*n* = 4310), *ABCA7* (*n* = 274), and *ABCA4* (*n* = 2224) harbored the highest number of variants, whereas > 10-fold less variations were found in *ABCD1* (*n* = 496), *ABCE1* (*n* = 407), and *ABCB7* (*n* = 271; Fig. 1d). However, when the number variants were normalized by gene length, no significant differences were identified between the subfamilies (Supplementary Figure 1A). In contrast, variability varied more than sevenfold between different *ABC* genes with *ABCB9* (*n* = 802.4 variants/kb) and *ABCB8* (*n* = 537.4 variants/kb) being most polymorphic, whereas *ABCB7* was most invariant (*n* = 120.1 variants/kb; Supplementary Figure 1B). To directly compare the evolutionary constraint, we compared the observed number of missense and loss-of-function variants in *ABC* genes with the expected numbers based on the genetic background variability. Missense variations in *ABCC9*, *ABCA2,* and *ABCE1* were most depleted, whereas, surprisingly, *CFTR* was least conserved and harbored 30% more missense variations than expected by chance (Supplementary Figure 2A; Supplementary Table 1). Based on genetic constraints on loss-of-function variations, 4 genes, including *ABCA2* and *ABCE1*, as well as *ABCB7* and *ABCD1* were considered as haploinsufficient, whereas little constraint on loss-of-function variations was detected in the remaining 44 *ABC* transporters (Supplementary Figure 2B; Supplementary Table 1).

In addition to SNVs, 46 of the 48 *ABC* transporter genes (96%) harbored CNVs, in which multiple exons up to the entire were deleted or duplicated (Fig. 1e). Overall, most CNVs were detected for *ABCC6* (230 CNVs), *ABCC1* (178 CNVs), and *ABCA6* (81 CNVs), whereas no CNVs were identified in *ABCB7* and *ABCD1*. While these CNVs are very likely to result in functional alterations, all deletions and duplications were found to be very rare with minor allele frequencies < 0.1%.

### Worldwide frequencies of human *ABC* transporter polymorphisms with putative clinical relevance

Next, we systematically analyzed the global and population-specific frequencies of clinically important variants in *ABC* transporters linked to drug response or ADR risk. Specifically, we considered all variants as putatively clinically relevant for which an association with drug-response phenotypes or related traits, such as overall or disease-specific survival upon chemotherapy, have been reported. In *ABCB1,* we assessed the population frequencies of 10 SNPs (Table 1). The missense variant rs2032582 and the synonymous polymorphisms rs1045642 constitute arguably the most extensively studied *ABCB1* variants and have been associated with risk of adverse reactions upon fluoropyrimidine therapy (Gonzalez-Haba et al. 2010) as well as toxicity to taxanes (Kim et al. 2012b) and anthracyclines (Ji et al. 2012; Wu et al. 2012). These variants are in strong linkage disequilibrium (Horinouchi et al. 2002) and have been shown to be associated with altered mRNA levels and protein folding (Cascorbi 2006). Rs2032582 constitutes a triallelic variant of amino acid position 893 with the reference sequence encoding an alanine and variants giving rise to a serine or threonine, respectively (Supplementary Figure 3). Ala893 is the predominant allele in Africans and East Asians, whereas in South Asians, Ser893 is most abundant (frequency 60.9% compared to 34.8% for Ala893). Thr893 is less prevalent ranging in frequencies between 0.4% in Africans and 13.3% in East Asians. Further *ABCB1* variants of clinical relevance are the missense variants rs2229109 and rs9282564, which are associated with increased risk of relapse of acute lymphoblastic leukemia (Gregers et al. 2015) and paclitaxel toxicity (Bergmann et al. 2012), respectively. Both variants are most frequently found in Europeans (MAF = 4.3% and 10.8%) and least prevalent in Africans (MAF = 0.7% and 1.6%) and East Asians (MAF = 0 and < 0.1%). Linkage analyses revealed one haplotype block of four SNPs (rs1128503, rs4148737, rs12720066 and rs1045642) with moderate-linkage disequilibrium, which could have potentially important implications for clinical associations of these variants (Supplementary Figure 4A).

**Table 1:** Population-specific frequencies of clinically important *ABCB1* (MDR1; P-gp) variants

Variant	Type	Minor allele frequencies (in %)						Clinical association of the minor allele	Effect or statistic	References	Sample size
		EUR	AFR	EAS	SAS	AMR	AJ				
rs2032582 (triallelic)	Missense (A893S or A893T)	A: 54.7; S: 41.8; T: 3.5	A: 91.8; S: 7.8; T: 0.4	A: 47.8; S: 38.9; T: 13.3	A: 34.8; S: 60.9; T: 4.3	A: 54.6; S: 40; T: 5.4	A: 62.4; S: 35; T: 2.6	Major molecular response to imatinib in chronic myeloid leukemia	78% of carriers achieved major molecular response versus 47.1% of non-carriers	Dulucq et al. (2008)	86
								Toxicity of taxanes and platinum compounds in ovarian cancer patients	OR = 3.1 and 9.7 for hematological and gastrointestinal toxicity, respectively	Kim et al. (2009b)	108
								PFS in gastric cancer patients treated with paclitaxel	HR = 2.6	Chang et al. (2010)	43
								OS in in metastatic colorectal cancer patients receiving first-line FOLFIRI treatment	12-month survival of 78% in variant carriers compared to 70% in controls	De Mattia et al. (2013)	250
								Toxicity of induction chemotherapy (idarubicin plus cytarabine) in acute myeloid leukemia	OR = 2.9 and 5.1 for hepatic and renal ADRs, respectively	Megías-Vericat et al. (2017)	221
								Decreased response to modafinil in narcolepsy patients	OR = 0.28	Moresco et al. (2016)	107
rs1128503	Synonymous	43.3	18.9	64.3	60.7	49.1	36.7	Toxicity of capecitabine in colorectal cancer patients	OR = 4.3 and 5.3 for neutropenia and HFS, respectively	Gonzalez-Haba et al. (2010)	54
								Major molecular response to imatinib in chronic myeloid leukemia	85% of hom carriers achieved major molecular response versus 47.7%	Dulucq et al. (2008)	85
								Toxicity of gefitinib in advanced NSCLC patients	OR = 15.8 and 10.8 for skin rash and diarrhea, respectively	Ma et al. (2017)	59
								Toxicity of induction chemotherapy (idarubicin plus cytarabine) in acute myeloid leukemia	OR = 6.9 and 3.8 for hepatic and renal ADRs, respectively	Megías-Vericat et al. (2017)	225
								Decreased response to FEC breast cancer chemotherapy	OR = 4.6	Chaturvedi et al. (2013)	100
								Decreased toxicity to FEC breast cancer chemotherapy	OR = 1.9 for grade 2–4 toxicity	Chaturvedi et al. (2013)	200
								Decreased response to modafinil in narcolepsy patients	OR = 0.31	Moresco et al. (2016)	107
rs2229109	Missense (S400N)	4.3	0.7	0	1.5	1.7	2.8	Increased risk of relapse of acute lymphoblastic leukemia patients to chemotherapy	OR = 2.9 of carriers vs. controls	Gregers et al. (2015)	518
rs1045642	Synonymous	53.4	20	36.7	39.5	45.4	35.6	Bone marrow toxicity during doxorubicin, vincristine and prednisolone induction therapy	*p* = 0.01 (control vs. het) and *p* < 0.0001 (control vs. hom)	Gregers et al. (2015)	517
								Increased exposure and toxicity of methotrexate in acute lymphoblastic leukemia or non- Hodgkin lymphoma patients	OR = 2.5 and 8.6 of carriers for plasma levels and hepatic toxicity, respectively	Suthandiram et al. (2014)	71
								PFS in gastric cancer patients treated with paclitaxel	HR = 4.6	Chang et al. (2010)	43
								Protective effect on arthralgia upon anastrozole therapy in postmenopausal breast cancer patients	OR = 0.3	Gervasini et al. (2017)	78
								Increased response to modafinil in narcolepsy patients	OR = 0.21 when comparing hom vs het carriers	Moresco et al. (2016)	107
rs9282564	Missense (N21D)	10.8	1.6	< 0.1	2.4	2.9	2.4	Associated with toxicity of paclitaxel and carboplatin therapy in ovarian cancer patients in exploratory analysis	*p* = 0.03	Bergmann et al. (2012)	92
								Decreased serum tacrolimus levels after kidney transplantation	*p* = 0.001	Hu et al. (2018)	163
rs3213619	5′ UTR	4.0	8.4	3.8	N.A	4.7	3.0	Decreased risk of neuropathies in breast cancer patients treated with paclitaxel	OR = 0.47	Abraham et al. (2014)	1303
								Increased atenolol efficacy	*p* = 0.0002	McDonough et al. (2013)	768
rs12720066	Intron	5.5	1	0	N.A	3.5	5.2	Decreased risk of irinotecan-induced neutropenia	β = 0.286	Li et al. (2018b)	78
rs4148737	Intron	42.9	44.9	28.9	N.A	41.4	48.6	Reduced OS of osteosarcoma patients after chemotherapy	HR = 3.7 per allele	Caronia et al. (2011)	91
rs3842	3′UTR	13.6	16.9	26.2	N.A	14.2	17.5	Increased clearance of efavirenz in HIV-1 patients	*p* = 0.001	Mukonzo et al. (2014)	99
rs10267099	Intron	77.1	83.2	99.7	N.A	86.3	73.5	Decreased response to atenolol	*p* = 0.006	McDonough et al. (2013)	768

*PFS* progression-free survival, *OS* overall survival, *OR* odds ratio, *EUR* Europeans, *AFR* Africans, *EAS* East Asians, *SAS* South Asians, *AMR* Latinos, *AJ* Ashkenazi Jews, *N.A.* not available

In the *ABCC* subfamily, we analyzed the population-specific frequencies of 25 SNVs that were correlated with chemotherapy outcomes or toxicity (Table 2). Interestingly, frequencies of risk variants for anthracycline-induced cardiotoxicity (ACT) were highly population-specific and differed > 100-fold between populations. The cardioprotective synonymous variant rs246221 in *ABCC1* (Semsei et al. 2012) was most common with frequencies between 20.3% and 65.2% in South Asians and Africans, respectively. By contrast, East Asians did not harbor the risk variants rs8187710 (*ABCC2*) and rs45511401 (*ABCC1*), which are common in all other populations with frequencies up to 5.6% and 15.7%, respectively. Notably, rs45511401 is in linkage disequilibrium with the intronic ACT risk variant rs4148350 (*R*^2^ = 0.153; Supplementary Figure 4B), indicating that both associations might to some extent be traced back to the same genetic signal.

**Table 2:** Population-specific frequencies of clinically important variants in genes of the *ABCC* subfamily

Variant	Type	Minor allele frequencies (in %)						Clinical association of the minor allele	Effect or statistic	References	Sample size
		EUR	AFR	EAS	SAS	AMR	AJ				
***ABCC1 (MRP1)***											
rs45511401	Missense (G671V)	5.6	1.2	< 0.1	1.6	1.7	3.3	Increased risk of anthracycline-induced cardiotoxicity	OR = 3.6	Wojnowski et al. (2005)	42
rs4148350	Intron	7.3	10.4	4.0	N.A	8.4	6.6	Increased risk of anthracycline-induced cardiotoxicity	OR = 3.4	Visscher et al. (2012)	156 and 188 and 96
								Toxicity of induction chemotherapy (idarubicin plus cytarabine) in acute myeloid leukemia	OR = 5.3 for grade 3–4 hepatic toxicity	Megías-Vericat et al. (2017)	225
rs246221	Synonymous	30.5	65.2	42.5	20.3	35.2	32.1	Decreased risk of anthracycline-induced cardiotoxicity	Increased LVFS of homozygous carriers (40.7%) compared to het and controls (38.4%)	Semsei et al. (2012)	164
rs3743527	3′ UTR	22.6	14.3	45.7	N.A	27.0	23.8	Increased risk of anthracycline-induced cardiotoxicity	Decreased LVFS of homozygous carriers (34%) compared to het and controls (39.3%)	Semsei et al. (2012)	168
rs17501331	Intron	10.3	2.2	0	N.A	5.8	11.7	Increased risk of irinotecan-induced neutropenia	β = − 0.295	Li et al. (2018b)	78
rs212091	3′UTR	14.9	13.0	25.4	N.A	10.2	9.7	Virological failure of protease inhibitor regimens in HIV patients	OR = 4.4	Coelho et al. (2013)	87
rs119774	Intron	6.8	1.6	0.3	N.A	4.4	6.9	Increased response to montelukast in asthma	*p* = 0.004 when comparing hom vs het carriers	Lima et al. (2006)	49
rs2074087	Intron	84.4	81.5	82.5	68.2	78.9	77.0	Increased risk of azathioprine-induced lymphopenia	OR = 3.4	Lee et al. (2015)	131
***ABCC2 (MRP2)***											
rs8187710	Missense (C1515Y)	5.6	15.7	< 0.1	1.9	4.1	12.9	Increased risk of anthracycline-induced cardiotoxicity	OR = 2.3	Wojnowski et al. (2005)	44
rs3740065	Intron	9.7	21.6	34.1	N.A	14.0	17.2	Response of patients with invasive breast cancer to tamoxifen monotherapy	HR = 10.6	Kiyotani et al. (2010)	282
rs3740066	Synonymous	37	25.9	23	32.6	34.9	34.5	Severe toxicity of irinotecan in NSCLC patients	OR = 5.6	Han et al. (2009)	107
rs12762549	Intergenic	46.8	43.0	56.5	N.A	49.3	51.7	Leukopenia risk upon docetaxel therapy	OR = 3.1	Kiyotani et al. (2008)	113
								Response of NSCLC patients to second line docetaxel therapy	OR = 7.3	Szczyrek et al. (2017)	52
rs717620	5′ UTR	19.9	5.8	21.4	11.3	13.0	20.0	Poor response and reduced OS of SCLC patients undergoing etoposide and/or platinum-based therapy	HR = 2.1 and 1.9 for response and OS, respectively	Campa et al. (2012)	167 and 127
rs17222723	Missense (V1188E)	5.6	6.0	< 0.1	1.8	3.6	12.8	Increased response of esophageal cancer patients to platinum-based therapy	OR = 0.21 of carriers compared to controls	Rumiato et al. (2016)	116
***ABCC3 (MRP3, MOAT-D)***											
rs1051640	Synonymous	18.2	8.6	5.6	10.3	8.6	18.7	Increased risk of cisplatin-induced hearing loss	OR = 1.8	Pussegoda et al. (2013)	247
rs4148416	Synonymous	5.4	19.6	15.0	8.8	14.4	5.8	Reduced OS of osteosarcoma patients after chemotherapy	HR = 8.1 per allele	Caronia et al. (2011)	91
								Poor response to chemotherapy in osteosarcoma patients	OR = 3.8	Yang et al. (2013)	45
rs4148405	Intron	14	43.7	21.7	N.A	21.8	21	Shorter disease-free survival in acute myeloid leukemia patients treated with cytarabine and etoposide	HR = 3.2	Yee et al. (2013)	153
***ABCC4 (MRP4, MOAT-B)***											
rs2274405	Synonymous	34.1	30.3	47.1	36.1	42.7	46.4	Increased response of esophageal cancer patients to platinum-based therapy	OR = 0.56 and 0.15 of het and hom carriers, respectively, compared to controls	Rumiato et al. (2016)	116
***ABCC5 (MRP5, MOAT-C)***											
rs3749438	3′ UTR	36.1	26.2	40.6	N.A	28.4	39.7	Increased risk of irinotecan-induced severe toxicity in metastatic colorectal cancer patients	OR = 1.9–2.1	Chen et al. (2015a)	452 and 322
rs10937158	Intron	54.4	74.7	86.0	N.A	51.0	53.3	Decreased risk of irinotecan-induced severe toxicity in metastatic colorectal cancer patients	OR = 0.4–0.45	Chen et al. (2015a)	328 and 448
rs7627754	Promoter	11.4	36.0	34.9	N.A	18.5	7.6	Increased risk of doxorubicin-induced cardiotoxicity	8–12% reduction of ejection and shortening fractions of hom carriers	Krajinovic et al. (2016)	251
rs7636910	Synonymous	36.9	26.8	41.5	36.4	26.9	40.9	Increased response and OS in pancreatic adenocarcinoma patients treated with gemcitabine-based chemoradiotherapy	OR = 1.7	Tanaka et al. (2011)	261
***ABCC6 (MRP6, MOAT-E)***											
rs2238472	Missense (R1268Q)	28.2	10.1	12.3	18.2	31.3	17.0	Increased toxicity of docetaxel and thalidomide in castration-resistant prostate cancer patients	*p* = 0.006	Deeken et al. (2009)	47
***ABCC10 (MRP7)***											
rs2125739	Missense (I948T)	25.1	31.9	10.9	18.0	18.7	21.3	Associated with nausea of paclitaxel and carboplatin therapy in ovarian cancer patients in exploratory analysis	*p* = 0.002	Bergmann et al. (2012)	92
								Increased OS in CRC patients who received oxaliplatin- based chemotherapy	OR = 0.56	Kap et al. (2016)	623
***ABCC11 (MRP8)***											
rs17822931	Missense (G180R)	13	2.8	87	40.6	16.2	10.8	Reduced MRP8 expression and increased disease-free survival upon nucleoside-based chemotherapy	*p* < 0.03	Guo et al. (2009) and Uemura et al. (2010)	/

*CRC* colorectal cancer, *OR* odds ratio, *LVFS* left-ventricular fraction shortening, *OS* overall survival, *NSCLC* non-small cell lung cancer, *EUR* Europeans, *AFR* Africans, *EAS* East Asians, *SAS* South Asians, *AMR* Latinos, *AJ* Ashkenazi Jews, *N.A.* not available

Multiple *ABCC* variants associated with irinotecan (rs3740066 in *ABCC2*, rs4148405 in *ABCC3* as well as rs3749438 and rs10937158 in *ABCC5*) or taxane (rs12762549 in *ABCC2* as well as rs2238472 and rs2125739 in *ABCC6*) toxicity or response were overall less population-specific and differed only by < 3-fold across populations with the exception of rs17501331 in *ABCC1*, which was not identified in East Asians (MAF = 0%) but reached frequencies of 11.7% and 10.3% in Ashkenazim and Europeans. By contrast, variants associated with response to platinum-based therapy differed substantially between ethnicities, including rs717620 (MAF between 21.4% in East Asians and 5.8% in Africans), rs17222723 (MAF between 12.8% in Ashkenazi Jews and < 0.1% in East Asians), and rs1051640 (MAF between 18.7% in Jews and 5.6% in East Asians). MRP8 encoded by *ABCC11* is an export pump for nucleotide analogues (Oguri et al. 2007) and is associated with pemetrexed resistance (Uemura et al. 2010). The variant rs17822931 that results in proteasomal degradation of MRP8 (Toyoda et al. 2009) differs > 30-fold between populations with relatively low frequencies in Africans (MAF = 2.8%), whereas the variant constitutes the dominant genotype in East Asian populations (MAF = 87%).

The *ABCG2* gene, encoding the BCRP transporter, harbors two important missense polymorphisms, which have been consistently implicated in response and toxicity of TKIs (Table 3). Rs2231142 results in increased risk of gefitinib toxicity (Cusatis et al. 2006) and increased rates of major molecular response to imatinib (Jiang et al. 2017). Similar effects on response and overall survival were found for rs2231142 (Chen et al. 2015b; Kim et al. 2009a), which is not linked with rs2231137 (Supplementary Figure 4C). Notably, both variants were most prevalent in East Asian and Latin Americans, whereas their frequencies were substantially lower in all other populations analyzed. Only a few associations of pharmacological or toxicological phenotypes with genetic variants in *ABC* transporters beyond *ABCB1*, *ABCG2,* and the *ABCC* subfamily have been presented to date (Supplementary Table 2).

**Table 3:** Population-specific frequencies of clinically important variants in *ABCG2* (BCRP)

Variant	Type	Minor allele frequencies (in %)						Clinical association of the minor allele	Effect or statistic	Reference	Sample size
		EUR	AFR	EAS	SAS	AMR	AJ				
rs2231135	5′ UTR	7.0	2.0	0	N.A	4.3	4.0	Increased risk of mucositis in osteosarcoma patients treated with high-dose methotrexate	OR = 2.5 for grade 2–3 mucositis in carriers compared to control	Jabeen et al. (2015)	57
rs2231142	Missense (Q141K)	10.4	2.7	30.7	9.3	22.6	6.6	Increased diflomotecan exposure	Plasma levels increased threefold in het carriers compared to controls	Sparreboom et al. (2004)	22
								Gefitinib toxicity in NSCLC patients	OR = 5.7 for dose-limiting diarrhea	Cusatis et al. (2006)	124
								Higher rate of major molecular response to imatinib therapy (meta analysis)	OR = 0.65	Jiang et al. (2017)	2184
								Increased PFS of advanced stage ovarian cancer patients treated with platinum and taxane-based chemotherapy	22.7 months PFS in carriers versus 16.8 months in controls	Tian et al. (2012)	506
								Decreased response of allopurinol	*p* = 3.4 × 10^–7^	Wen et al. (2015)	2027
rs2231137	Missense (V12M)	4.1	6.6	32.8	14.0	23.7	10.5	Severe toxicity of irinotecan in NSCLC patients	OR = 5.1	Han et al. (2009)	107
								Improved response to imatinib therapy in chronic myeloid leukemia patients	OR = 0.64 for complete cytogenetic response in carriers compared to controls	Kim et al. (2009a)	229
								Longer OS in NSCLC patients receiving TKI therapy	31 months OS in carriers versus 18 months in controls	Chen et al. (2015b)	70
								Improved treatment outcomes in acute myeloid leukemia patients receiving cytarabine or anthracyclines	HR = 0.44 for OS	Hampras et al. (2010)	261
								Increased toxicity in acute myeloid leukemia patients receiving cytarabine or anthracyclines	OR = 8.4	Hampras et al. (2010)	261
rs7699188	Intron	15.6	44.1	7.6	N.A	13.2	23.1	Toxicity in in metastatic colorectal cancer patients receiving first-line FOLFIRI treatment	OR = 7.3	De Mattia et al. (2013)	250
rs3109823	Intron	71.8	44	78.8	N.A	80.6	83.4	Improved response and OS of SCLC patients undergoing etoposide and/or platinum-based therapy	OR = 0.3 and 0.6 for response and OS, respectively	Campa et al. (2012)	171
rs13120400	Intron	30.5	6.2	0	N.A	14.7	17.4	Increased blood concentration of deferasirox	OR = 4.1	Allegra et al. (2016)	Not reported
rs2199936	Intron	89.4	87.6	67.1	N.A	80.9	92.8	Decreased Increased response to rosuvastatin	Effect of + 5.2 mg/dl	Chasman et al. (2012)	6989
rs4148155	Intron	10.5	2.3	32.8	N.A	18.6	6.6	Increased response to allopurinol	*p* = 7.89 × 10^–9^	Brackman et al. (2019)	4446

*PFS* progression-free survival, *OR* odds ratio, *NSCLC* non-small cell lung cancer, *SCLC* small cell lung cancer, *TKI* tyrosine kinase inhibitor, *OS* overall survival, *EUR* Europeans, *AFR* Africans, *EAS* East Asians, *SAS* South Asians, *AMR* Latinos, *AJ* Ashkenazi Jews, *N.A.* not available

### Functional consequences of rare genetic variation in human *ABC* transporters

**Fig. 2:**
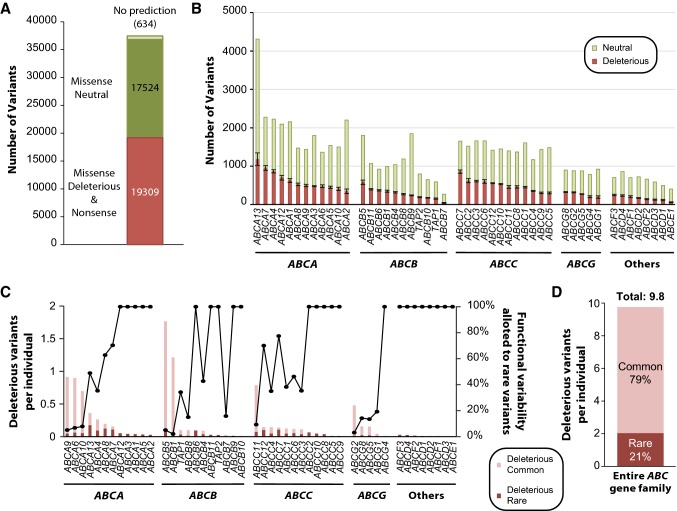
*ABC* transporter genes harbor a plethora of genetic variants with functional consequences, many of which are rare. **a** In total, 37,467 variants affected the amino acid sequence of the corresponding gene product (missense and frameshift variants, variants that resulted in gain of a stop or loss of a start codon or that affected splice sites) of which 19,309 were predicted to result in functional consequences. **b** The number of deleterious and functionally neutral variants differs drastically between *ABC* transporter genes. Error bars indicate standard error of the mean (SEM) across five computational algorithms (see methods for details). **c** The average number of deleterious variants per *ABC* transporter are shown per individual (stacked columns; left ordinate). Note that the relative importance of rare genetic variations with frequencies < 1% differs substantially between genes (indicated by black dots; right ordinate). Calculations consider a diploid human genome. **d** Overall, each individual was found to harbour on average 9.8 genetic variations in the *ABC* transporter superfamily that affect transporter function. Rare variants accounted for 21% of this genetically encoded functional variability

Next, we aimed to estimate the functional importance of rare *ABC* variations for which no experimental analyses or clinical association data were available. To this end, we used five partly orthogonal algorithms to predict the functional consequences. Of all 37,467 variants affecting the amino acid sequence of the encoded polypeptide, 19,309 variants (51.5%) were predicted to result in functional alterations of the respective *ABC* transporter (Fig. 2a; see methods). While functional effects can comprise both, variations that result in increased or decreased transporter function, previous studies showed that computational algorithms are significantly better at predicting loss-of-function effects compared to gain-of-function effects (Flanagan et al. 2010). We thus refer to variants with putative functional impacts as “deleterious” throughout this manuscript; however, we would like to alert the reader that the inclusion of some variants that result in increased transporter function cannot be excluded. Most deleterious variants were found in *ABCA13* (*n* = 1183), *ABCA7* (*n* = 953), and *ABCA4* (*n* = 865), whereas *ABCE1* (*n* = 60) and *ABCB7* (*n* = 43) harbored least (Fig. 2b). The multi-drug resistance transporters *ABCB1* (*n* = 344), *ABCC1* (*n* = 453), and *ABCG2* (*n* = 315) harbored medium numbers of variants with functional consequences.

Notably, only 14.8% (30 of 203) of common *ABC* missense variants with MAF > 1% were putatively deleterious, compared to 45.7% (15,152 of 33,137) for rare variations. The burden of functional genetic variability differed drastically between genes with an average diploid human genome harboring on average 1.8 and 1.2 variants with functional effects in *ABCB5* and *ABCB1*, respectively, whereas 29 transporters were highly conserved with < 0.1 functional variants per individual genome (Fig. 2c). In some transporters, including *ABCB1* and *ABCG2*, rare variations explained less than 10% of the genetically encoded functional variability. In contrast, rare variants are estimated to account for all variants with functional consequences in half (24 out of 48) of all human *ABC* transporter genes. Interestingly, the fraction of genetically encoded functional variability correlated significantly with the genetic constraint on the respective genes (*r* = 0.4; *p* = 0.005), suggesting that high evolutionary pressure tends to select against common variations that alter ABC transporter function. Overall, each individual was found to harbour 9.8 variants in the *ABC* gene family that entail functional alterations, of which 21% were attributed to by rare genetic variants (Fig. 2d).

### Genetic *ABC* transporter variability is highly population-specific

**Fig. 3:**
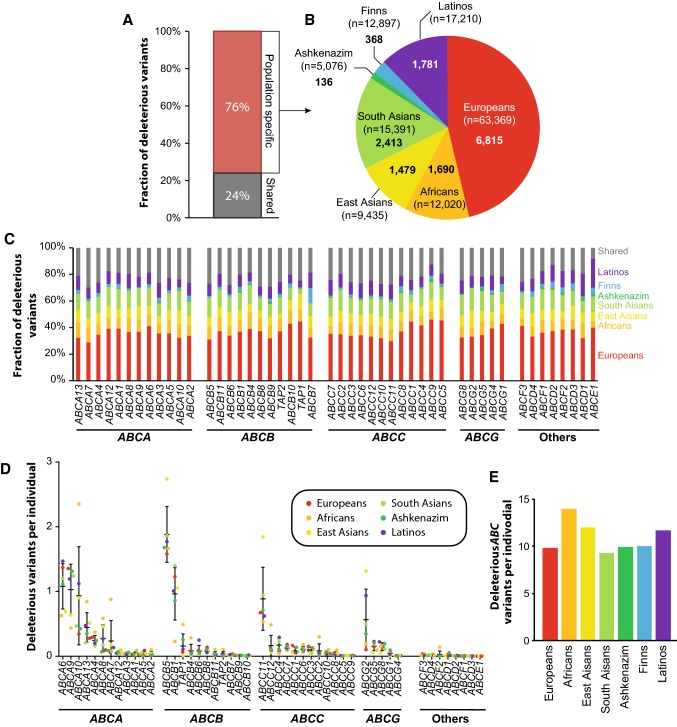
The genetically encoded functional variability of *ABC* transporters is highly population-specific. **a** The majority of genetic variations (76%) with putative functional impacts on ABC transporter function are population-specific. **b** Most of these population-specific variations were identified in Europeans. Numbers in bold indicate the total number of identified population-specific variations, while numbers in brackets denote the number of sequenced individuals for the respective population. **c** Stacked column plot showing the fraction of putatively functional variants specific to Europeans (red), Africans (orange), East Asians (yellow), South Asians (light green), Ashkenazi Jews (dark green), Finns (blue), and Latinos (purple). The fraction of variations that are found in at least two populations are shown in grey. **d** The number of *ABC* variants with functional consequences per individual is shown across populations. **e** Column plot depicting the functional *ABC* transporter variability when all putatively deleterious *ABC* transporter variants are aggregated. Note that African individuals harbour most functionally relevant *ABC* variants per individual, whereas functional variability in South Asians was overall lowest

The genetic landscape of the *ABC* transporter superfamily differed considerably between human populations. Of the putatively deleterious variants, only 24% were shared between two or more ethnicities, whereas 76% were population-specific (Fig. 3a). Most population-specific variants were found in Europeans (6815), whereas least were found in Ashkenazim (136). These differences are likely, at least in part, due to the unequal distribution of available sequencing data and the differences in genetic heterogeneity between the populations (Fig. 3b). The ratios of population-specific variants differed between ABC genes from 70% in *ABCA7* to 92% in *ABCE1*, whereas only 0.3% of variants were shared between all seven populations (Fig. 3c).

The observed population specificity is estimated to translate into inter-ethnic differences in *ABC* transporter function. The largest differences in variants with putative functional impacts across populations were identified for *ABCA10* where Africans harbor 2.4 putatively functional variations per individual compared to 0.3 in Europeans (Fig. 3d). Similar differences were observed for the breast cancer risk gene *ABCC11* (1.8 in East Asians compared to 0.5 in Africans), as well as the multi-drug resistance genes *ABCB1* (1.4 in South Asians compared to 0.2 in Africans) and *ABCG2* (1.3 in East Asians compared to 0.1 in Europeans). In contrast, inter-ethnic variability in *ABCC1* was less pronounced (0.16 in Europeans compared to 0.02 in East Asians). Overall, across the entire *ABC* transporter family Africans harbored most variations with putative functional impacts (13.9 deleterious variants per individual), whereas least variations were observed in South Asians (9.3 deleterious variants per individual; Fig. 3e).

### Structural consequences of genetic ABC variability

**Fig. 4:**
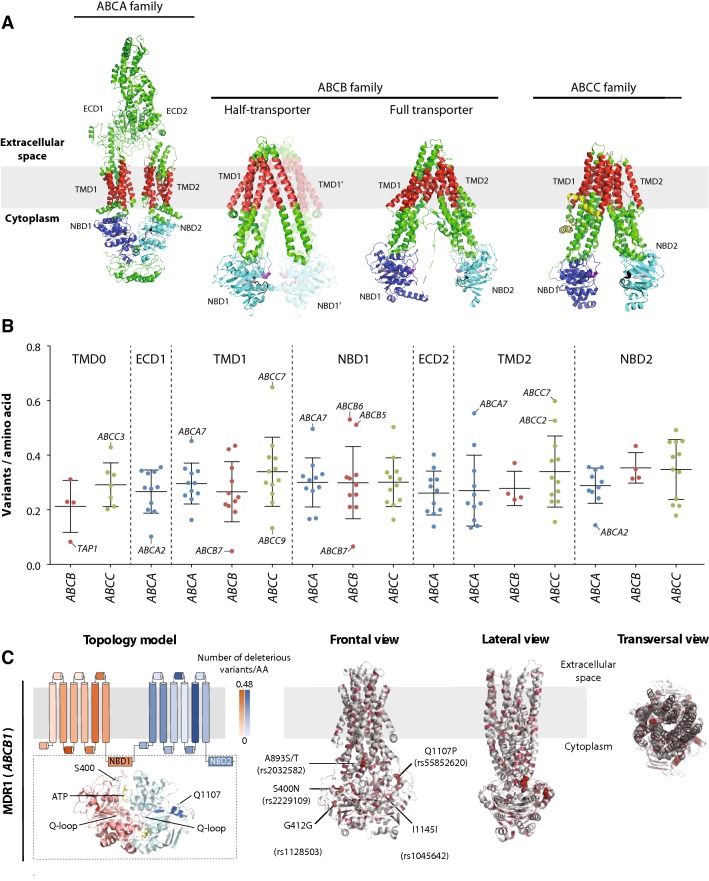
Structural analysis of putatively deleterious genetic variants of *ABC* transporter superfamily. **a** Illustration of the tertiary structures of ABCA, ABCB, and ABCC transporters. As representative examples, the structures of ABCA1 (PDB identifier 5XJY), ABCB10 (ABCB half transporter; PDB identifier 4AYT), ABCB11 (BSEP; ABCB full transporter), and ABCC7 (CFTR; PDB identifier 5UAK) are shown. Transmembrane domains (TMDs) are shown in red, nucleotide-binding domains (NBDs) are depicted in blue and turquoise, Walker motifs are colored in salmon and the N-terminal Lasso motif is depicted in yellow. **b** Overview of the genetically encoded structural variability stratified by ABC subfamily and domain. **c** Schematic topology models as well as 3D protein structures of MDR1 encoded by *ABCB1*. Different domains in the topology models are shaded based on the identified number of deleterious variants per amino acid in the respective domain. MDR1 constitutes two pseudo-symmetrical TMDs and NBDs encoded in a single polypeptide, colored in orange and blue, respectively. Detailed 3D structure of key protein domains with functionally relevant variants (sticks in cyan or magenta) and substrates (sticks in yellow) are shown as insets under the topology model. In the 3D model, all putatively deleterious variants with MAF > 0.1% are shown as light red spheres, whereas the corresponding part of the secondary structure motif is highlighted in salmon in case of variants with MAF < 0.1%. Note that N21D localizes to the lasso motif for which no crystallographic data were available and the variant is thus not shown. *ECD* extracellular domain, *TMD* transmembrane domain, *NBD* nucleotide-binding domain

Next, we characterized the distribution of genetic variability across ABC transporter domains by mapping the identified genetic variants onto the tertiary structures of the respective. We used experimentally determined crystal structures for all transporters of the ABCA, ABCB, and ABCC families for which such information was available (*n* = 18), while the remaining 16 structures were predicted using homology modeling. Typical ABC transporters consist of two α-helix transmembrane domains (TMDs) and two cytoplasmic nucleotide-binding domains (NBDs) that catalyse ATP hydrolysis (Fig. 4a). In addition to this backbone, some transporters have additional domains. ABCA transporters have two large extracellular domains (ECDs), while transporters of the ABCB and ABCC subfamilies contain an additional N-terminal TMD0 domain with unclear functional relevance. Furthermore, seven *ABC* genes of the *ABCB* subfamily encode only half-transporters (one NBD and one TMD domain) that require homo- or heterodimerization for transporter activity.

When stratifying by domains, we found that genetic variability differed substantially between transporters (Fig. 4b). The lowest numbers of variants per residue were found in the TMD0 domains of ABCB transporters with 0.21 variants/amino acid. In contrast, the NBD2 domains of ABCB and ABCC transporters are more variable (0.35 variants/amino acid). For individual genes, the TMD1 (0.05 variants/amino acid) and NBD1 domains (0.07 variants/amino acid) of ABCB7 were most conserved, while the TMD1 and TMD2 domains of ABCC7 (0.65 variants/amino acid) and ABCA7 (0.56 variants/amino acid), respectively, were > 10-fold more variable.

Finally, we aimed to corroborate our computational variant predictions using structural mapping approaches by focussing on the pharmacogenetically most important ABC transporter, MDR1 (also known as P-gp; encoded by *ABCB1*), for which high-resolution crystal structures are available (Kim and Chen 2018) (Fig. 4c). The clinically important missense variation A893S/T is located in the second intracellular loop of TMD2, which interacts with NBD1, and is necessary for structural stability. The S400N polymorphism is localized directly adjacent to the critical tyrosine at position 401, which coordinates the ATP in its binding pocket in NBD1 by direct van-der-Waals interactions with the adenine of the bound ATP molecule. Q1107P resides within the NBD2 Q-loop, which is necessary for ATPase activity and stabilizes the NBD dimer. No common variants were identified in any transmembrane helix or extracellular domain. However, we found a variety of rare variations in structurally important residues, including variants at the catalytic glutamate residue 556, which is required for ATP hydrolysis (Sauna et al. 2002), as well as various amino acid exchanges in the functionally critical NBD1 and NBD2 Q-loops (Zolnerciks et al. 2014).

### Ethnogeographic distribution of pathogenic *ABC* alleles can inform about Mendelian disease epidemiology

**Fig. 5:**
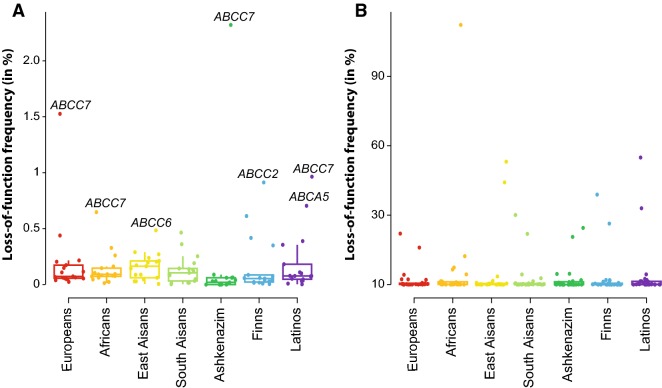
Genetic variability in *ABC* genes associated with genetic disorders can inform about population-specific disease risk. The gene-wise aggregated frequencies of loss-of-function (LoF) variants (frameshifts, start-lost, stop-gain, and splice site variants) are shown for *ABC* genes with known associations with congenital diseases (**a**) as well as for non-disease-associated genes (**b**)

We previously showed that the frequency of loss-of-function variants in *SLC* transporter genes implicated in recessive Mendelian disorders are suitable proxies to estimate population-specific disease risk (Schaller and Lauschke 2019). Here, we analyzed whether similar associations could be identified for *ABC* transporter genes. To this end, we comparatively analyzed the frequencies of loss-of-function variants, defined as frameshifts, start-lost or stop-gain variations or variants that affected critical splice site residues, in *ABC* transporter genes with or without implication in hereditary disease (Fig. 5).

Overall, 17 of 48 *ABC* genes are linked to autosomal recessive Mendelian disorders (Supplementary Table 3). Reduced CFTR (*ABCC7*) function is associated with cystic fibrosis (CF; OMIM 219700). We calculated homozygosity frequencies for *ABCC7* loss-of-function variants of 1 in 1850 and 1 in 4300 in Ashkenazim and European individuals, whereas frequencies in individuals of Africans and Asian ancestry were 1 in 24,000 and < 1 in 40,000, respectively. Impaired function variants in *ABCC6* are associated with pseudoxanthoma elasticum (PXE; OMIM 264800). In our data set, we find the highest aggregated *ABCC6* loss-of-function frequency in individuals of East Asian ancestry (0.5%), resulting in estimates of affected individuals of 1 in 42,530. Similarly, high carrier rates were identified in Europeans (0.4%; 1 in 52,000) and Finns (0.4%; 1 in 82,000), whereas risk allele prevalence was significantly lower in all other populations. Congenital generalized hypertrichosis (OMIM 135400) is a rare disease with varying presentations and comorbidities that is speculated to be, at least in part, caused by loss of *ABCA5* function (DeStefano et al. 2014). While global prevalence rates have, to our knowledge, not been reported, the disease was originally described in individuals of Mexican ancestry (Pavone et al. 2015), aligning with our finding of highest *ABCA5* loss-of-function frequencies in Latino populations (0.7%; 1 in 20,500).

In conclusion, these data provide an overview of the frequency of *ABC* loss-of-function variants in the general population that can be used to estimate population-specific Mendelian disease risk, thus providing valuable information for epidemiological rare disease research and clinical geneticists.

## Discussion

The *ABC* superfamily of transporters is of importance for drug response and toxicity, and genetic rare disease research. ABC transporters translocate a wide spectrum of endogenous substrates and medications. Consequently, identification of *ABC* transporters that interact with a drug candidate constitutes a critical step in drug discovery and development (Benadiba and Maor 2016; Yee et al. 2018). Previous clinical studies implicated genetic germline polymorphisms in at least 12 *ABC* genes with risk of adverse drug reactions or altered chemotherapy efficacy (Tables 1, 2, 3 and Supplementary Table 2). In addition, genetic variations in 21 *ABC* genes are causative for Mendelian disorders. Therefore, understanding the genetic landscape of *ABC* transporters constitutes a potentially important area for the personalization of oncological therapy and risk allele epidemiological study of relevant Mendelian diseases.

In this study, we detected a total of 62,793 exonic variants, the vast majority (98.5%) of which are rare and functionally poorly understood. In addition to these single-nucleotide variants and indels, we identified 1003 *ABC* alleles in which at least one exon was deleted or duplicated. Notably, somatic *ABC* gene CNVs have been implicated in acquired drug resistance. Studies of drug-resistant cell lines derived from human neoplasms identified amplifications of at least 13 *ABC* transporter genes, including *ABCB1*, *ABCC1* and *ABCC4* (Yasui et al. 2004). Conversely, deletions of the multi-drug resistance transporters predicted response to neoadjuvant therapy in breast cancer patients (Litviakov et al. 2016). Notably, while drug resistance is primarily characterized by somatic amplification events, the majority of CNVs in our data set were deletions and it will be interesting to observe whether patients with germline deletions of pharmacologically important drug transporters are predisposed to favorable therapeutic responses using drugs, which are substrates of the deleted transporter.

There is an increasing body of evidence describing differences in drug response, ADRs and clinical outcomes from chemotherapy based on genetic differences between ethnic groups (Phan et al. 2011). For instance, Caucasian colon cancer patients were at significantly higher risk to develop diarrhea, nausea, vomiting, and stomatitis during adjuvant 5-fluorouracil-based chemotherapy compared to African Americans (McCollum et al. 2002). Moreover, the risk of dose-limiting ADRs due to taxanes or platinum therapy was significantly lower in Caucasian lung cancer patients compared to patients of Asian descent, whereas response rates consistently showed inverse correlations (Gandara et al. 2009; Lara et al. 2009, 2010). This variability is likely to be at least in part caused by differences in the allelic distribution for genes involved in the disposition of the respective chemotherapeutics.

Mounting evidence suggests that the targeted interrogation of candidate pharmacogenetic polymorphisms is not sufficient to accurately predict the drug response of a given patient (Lauschke and Ingelman-Sundberg 2016, 2018). Importantly, our previous data indicate that variant burden rather than allele status of specific *ABC* variants is a predictor of clinical outcomes, thus corroborating that NGS-based approaches can add value to personalized cancer prognostics (Xiao et al. 2020). One plausible interpretation of this observation is that multiple *ABC* variants with individually small-effect sizes act modulate bioavailability of orally administered substrates and/or intra-tumoral drug concentrations in concert, thereby impacting treatment efficacy. These findings have important implications for cancer pharmacogenomics and incentivize studies into the underlying mechanisms.

Interestingly, mapping of clinically impactful variants onto the 3D structure of MDR1 revealed a preferential localization in NBDs. Generally, the NBDs in MDR1 are highly conserved compared to the substrate-binding domains, indicating that NBDs might be more sensitive to functional alterations, whereas impacts of variations in the substrate-binding domain or translocation channel seem to be less pronounced (Wolf et al. 2011). The two synonymous variants indicated here (G412G and I1145I), although not resulting in amino acid exchange, have been suggested to affect transporter function by disrupting the cotranslational folding process via introduction of rare codons (Kimchi-Sarfaty et al. 2007). The triallelic variation at position A893, which localizes to a less conserved transmembrane helix, has not been reported to affect transporter function in vitro (Kimchi-Sarfaty et al. 2002). Thus, functional effects associated with this variant might be due to the strong linkage with G412G and I1145I (Fung and Gottesman 2009).

Overall, we found that the *ABC* transporter superfamily was highly population-specific and inter-ethnic variability is commensurate with other genetically diverse pharmacogene families, including *CYP*s (Zhou et al. 2017), *SLCO*s (Zhang and Lauschke 2019) and *UGT*s (Kaniwa et al. 2005). Overall, 74.9% of all variants that were predicted to affect the functionality of the respective ABC transporter were specific to a single population and the overall load of functional genetic variability differed considerable between the analyzed populations. Inter-ethnic variability was furthermore reflected in differences in population-specific prevalence of ABC-associated Mendelian diseases with autosomal recessive inheritance. For instance, frequencies of CF are around 1 in 2500–3500 newborns of Caucasian ancestry, whereas only 1 in 17,000 and 1 in 31,000 children of African and Asian ancestry are affected, which closely aligns with predictions based on loss-of-function carrier rates (1 in 1850 in Europeans, 1 in 24,000 in Africans, and < 1 in 40,000 in East Asians). Similarly, PXE has been reported to have a prevalence around 1 in 50,000 Dutch individuals (Kranenburg et al. 2019), compared to our estimates of 1 in 52,000 in Europeans based on *ABCC6* loss-of-function allele frequencies. Interestingly, *ABCC6* was also the *ABC* gene that was found to harbour most CNVs, which is aligned with the previous studies describing genomic deletions in this locus in PXE patients (Costrop et al. 2010; Katona et al. 2005). Combined, these data suggest that population-scale sequencing data provide an important tool to predict Mendelian ABC disease risk. Notably, however, this approach is only suitable for diseases in which heterozygous loss of gene function is phenotypically silent, thus excluding autosomal dominant or X-linked modes of inheritance. Taken together, our analyses revealed striking ethnogeographic differences in *ABC* variability profiles that might explain at least part of the observed variability in chemotherapy response and incidence of Mendelian disorders between populations. Furthermore, the population-scale genomic data set presented here promises to provide a powerful resource for the evaluation of genetic ABC disease epidemiology.

In summary, we comprehensively profiled the genetic variability of the human *ABC* transporter superfamily and revealed a surprising extent of rare and population-specific variations. Computational evaluations of the functional impacts of these variants indicate that these variants contribute considerably to the variability in *ABC* transporter function with potentially important consequences for chemotherapeutic treatment regimens. Thus, these data incentivize the consideration of sequencing-based genotypes for patient stratification, particularly in the current era of clinical trial globalization. Furthermore, we expect that a deeper understanding of the functional consequences of *ABC* transporter variability might be useful to improve public health strategies and flag patients at risk of not responding appropriately to treatment with ABC substrates.

## Electronic supplementary material

Below is the link to the electronic supplementary material.

Supplementary Figure 1: Genetic variability of *ABC* transporters after normalization for gene length. A, No significant differences were identified for the number of variations per gene length between ABC subfamilies (p>0.05). B, Stacked column plot depicting the genetic variability of all 48 human ABC transporters normalized by gene length (PDF 239 kb)

Supplementary Figure 2: Evolutionary constraints in the human ABC gene superfamily. A, The evolutionary constraint of missense variations is shown for all 48 human *ABC* genes. Higher Z scores indicate a depletion of missense variations within the respective gene compared to the genetic background variation, whereas scores <0 indicate that the gene is less constraint. B, The probability of being loss-of-function intolerant (pLI) is plotted for each human *ABC* gene. Note that only 4 genes are considered haploinsufficient, whereas the remaining 44 ABC genes are not depleted of their expected loss-of-function variation. Numerical conservation values and confidence intervals can be found in Supplementary Table 1. Constraint information was calculated and provided by (Lek et al. 2016) (PDF 366 kb)

Supplementary Figure 3: Inter-ethnic differences of the clinically important triallelic *ABCB1* variant rs2032582. Frequencies of the different nucleotide and amino acid variations for the rs2032582 polymorphism are depicted for six worldwide populations (PDF 345 kb)

Supplementary Figure 4: Linkage disequilibrium and haplotype type structure of *ABCB1*, *ABCC1* and *ABCG2* loci. Linkage disequilibrium (LD) maps are shown for clinically important variants in *ABCB1* (A), *ABCC1* (B) and *ABCG2* (C) are shown. LD is depicted as correlation between variant pairs (R2). Two weak haplotype blocks were identified for *ABCB1* and *ABCC1* (indicated by black frames), whereas the analysed variations in *ABCG2* were only in very weak LD (PDF 389 kb)

Supplementary Table 1: Overview of evolutionary constraint scores in the human *ABC* transporter family. o/e indicates the ratio of observed to expected variations. Values in brackets indicate the 90% confidence interval. pLI indicates the probability of being loss-of-function intolerant. Z scores indicate the deviation of observed counts from the expected variant number (XLSX 12 kb)

Supplementary file6 (DOCX 25 kb)

Supplementary Table 3: Overview of Mendelian disease associations and their respective mode of inheritance for all human *ABC* genes (XLSX 15 kb)
