# Supplementary figures and images for: Ethnogeographic and inter-individual variability of human ABC transporters
Source: Hum Genet. 2020 Mar 23;139(5):623–46. doi: 10.1007/s00439-020-02150-6 (PMC7170817; doi:10.1007/s00439-020-02150-6)

# Supplementary Figure 1

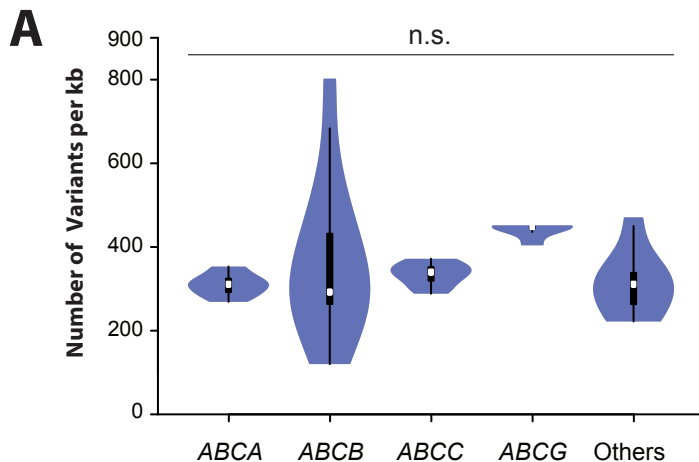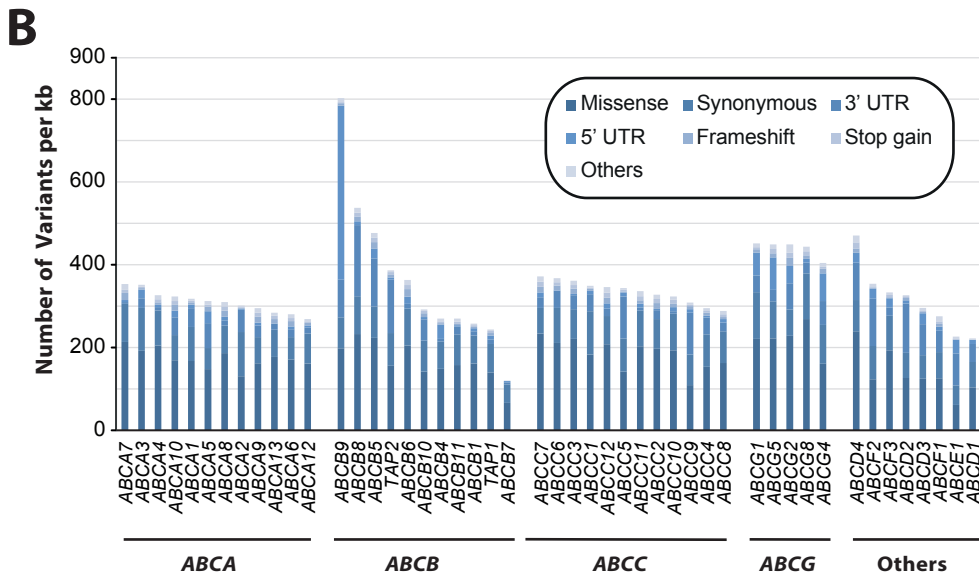

Supplement: Supplementary file 1 — Supplementary Figure 1: Genetic variability of ABC transporters after normalization for gene length. A, No significant differences were identified for the number of variations per gene length between ABC subfamilies (p>0.05). B, Stacked column plot depicting the genetic variability of all 48 human ABC transporters normalized by gene length (PDF 239 kb) [file 439_2020_2150_MOESM1_ESM.pdf]

# Supplementary Figure 2

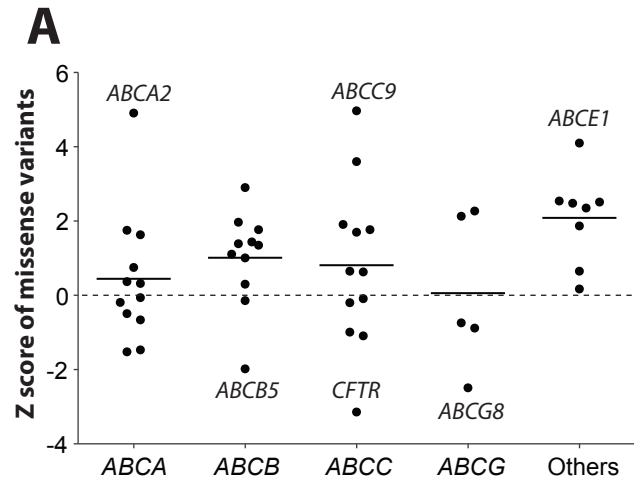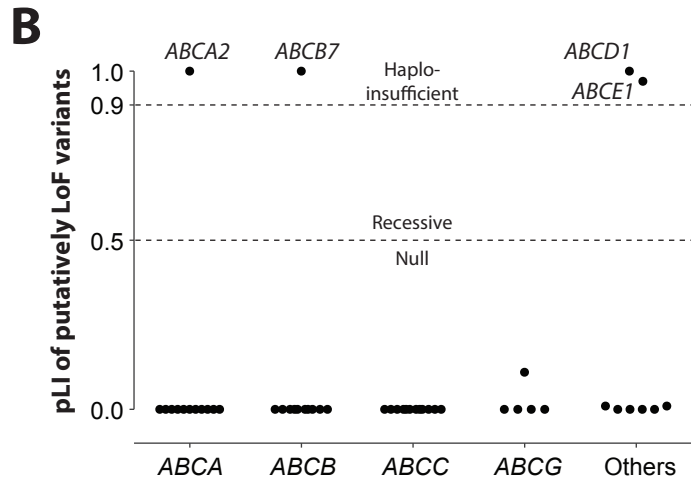

Supplement: Supplementary file 2 — Supplementary Figure 2: Evolutionary constraints in the human ABC gene superfamily. A, The evolutionary constraint of missense variations is shown for all 48 human ABC genes. Higher Z scores indicate a depletion of missense variations within the respective gene compared to the genetic background variation, whereas scores <0 indicate that the gene is less constraint. B, The probability of being loss-of-function intolerant (pLI) is plotted for each human ABC gene. Note that only 4 genes are considered haploinsufficient, whereas the remaining 44 ABC genes are not depleted of their expected loss-of-function variation. Numerical conservation values and confidence intervals can be found in Supplementary Table 1. Constraint information was calculated and provided by (Lek et al. 2016) (PDF 366 kb) [file 439_2020_2150_MOESM2_ESM.pdf]

## Supplementary Figure 3

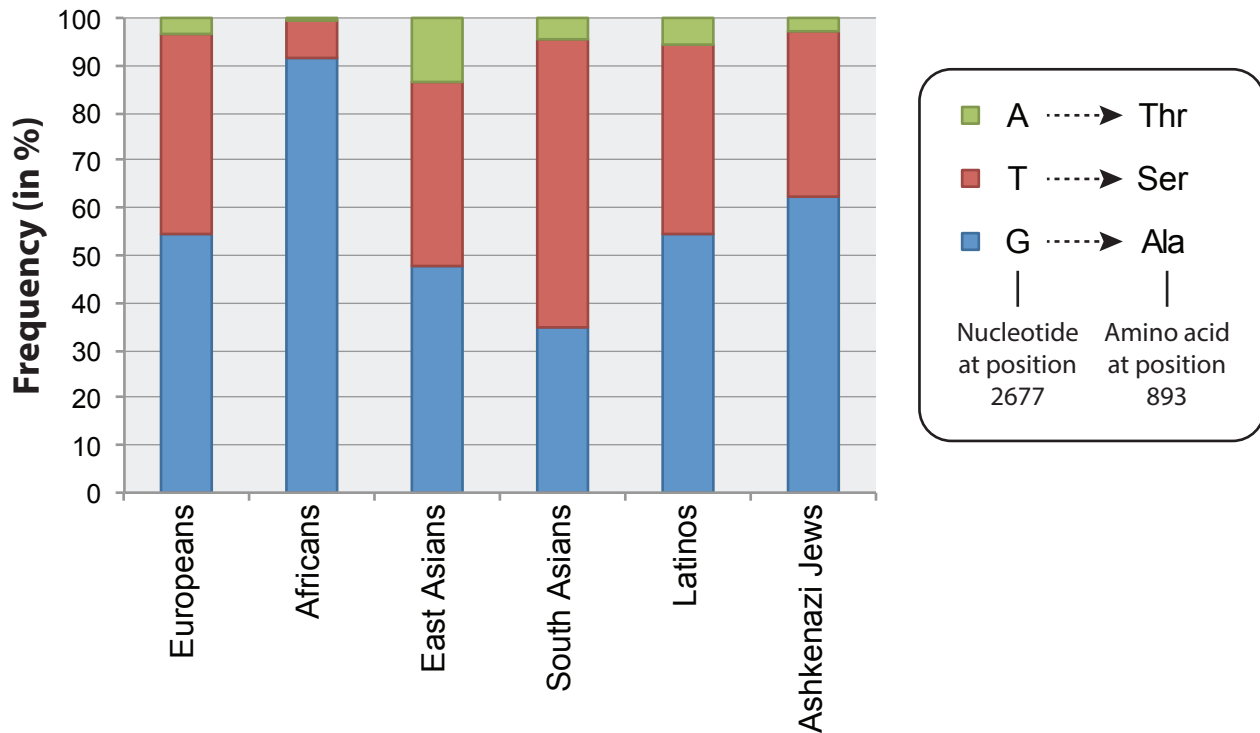

Supplement: Supplementary file 3 — Supplementary Figure 3: Inter-ethnic differences of the clinically important triallelic ABCB1 variant rs2032582. Frequencies of the different nucleotide and amino acid variations for the rs2032582 polymorphism are depicted for six worldwide populations (PDF 345 kb) [file 439_2020_2150_MOESM3_ESM.pdf]

Supplementary Figure 4

A

*ABCB1*

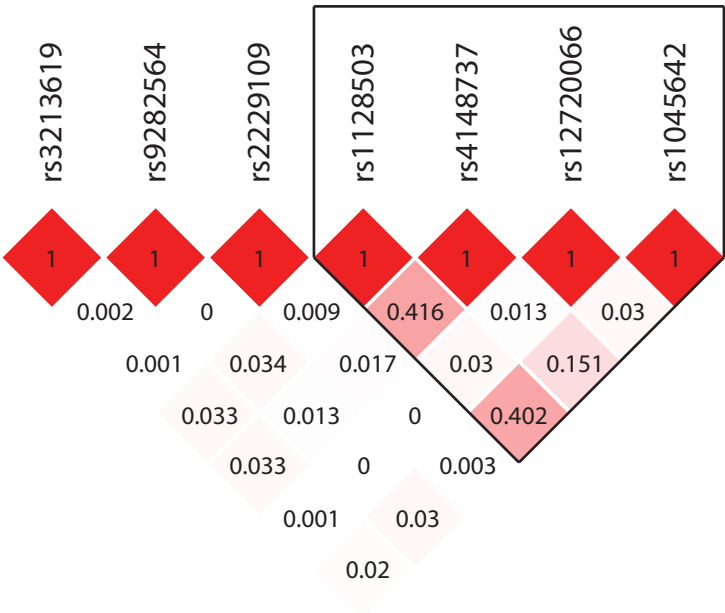

B

*ABCC1*

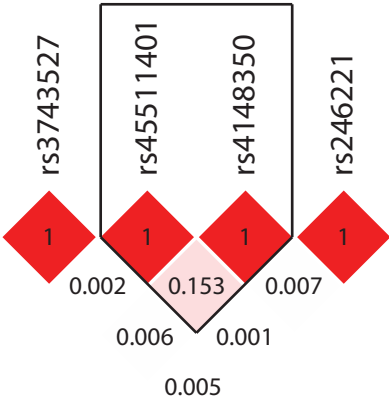

C

*ABCG2*

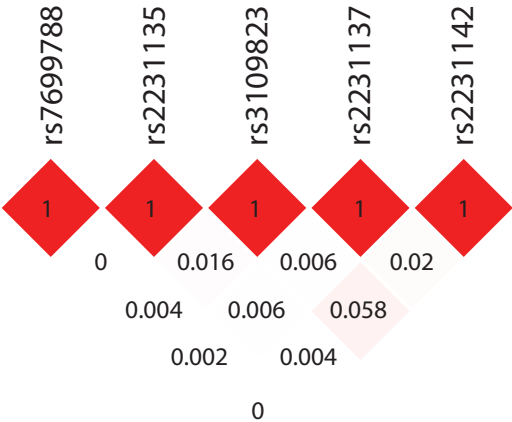

R²:

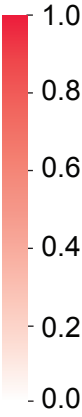

Supplement: Supplementary file 4 — Supplementary Figure 4: Linkage disequilibrium and haplotype type structure of ABCB1, ABCC1 and ABCG2 loci. Linkage disequilibrium (LD) maps are shown for clinically important variants in ABCB1 (A), ABCC1 (B) and ABCG2 (C) are shown. LD is depicted as correlation between variant pairs (R2). Two weak haplotype blocks were identified for ABCB1 and ABCC1 (indicated by black frames), whereas the analysed variations in ABCG2 were only in very weak LD (PDF 389 kb) [file 439_2020_2150_MOESM4_ESM.pdf]
