# Supplementary material for: Ethnogeographic and inter-individual variability of human ABC transporters
Source: Hum Genet. 2020 Mar 23;139(5):623–46. doi: 10.1007/s00439-020-02150-6 (PMC7170817; doi:10.1007/s00439-020-02150-6)
Supplement: Supplementary file 6 — Supplementary file6 (DOCX 25 kb) [file 439_2020_2150_MOESM6_ESM.docx]

**Supplementary Table 2: Population-specific frequencies of variants in other *ABC* genes with putative functional relevance.** EUR = Europeans; AFR = Africans, EAS = East Asians; SAS = South Asians; AMR = Latin Americans; AJ = Ashkenazi Jews; N.A. = not available.

| **Variant** | **Type** | **Minor allele frequencies (in %)** | | | | | | **Clinical association of the minor allele** | **Statistic** | **Reference** | **Sample size** |
| --- | --- | --- | --- | --- | --- | --- | --- | --- | --- | --- | --- |
|  |  | **EUR** | **AFR** | **EAS** | **SAS** | **AMR** | **AJ** |  |  |  |  |
| ***ABCA1*** | | | | | | | | | | |  |
| rs2230808 | Missense(K1587R) | 76.0 | 21.2 | 59.1 | 64.1 | 79.2 | 74.4 | Decreased HDL after fenofibrate treatment | p=0.004 | (Tsai et al. 2010) | 250 |
| rs2230806 | Missense(R219K) | 27.4 | 62.5 | 43.1 | 36.7 | 32.3 | 28.1 | Increased HDL after fenofibrate treatment | p=0.02 | (Tsai et al. 2010) | 287 |
| rs12003906 | Intron | 0.5 | 21.3 | <0.1 | 0.4 | 1.7 | 7.1 | Decreased statin-mediated LDLc reduction | p=0.0001 | (Voora et al. 2008) | 509 |
| ***ABCA5*** | | | | | | | | | | |  |
| rs536009 | Missense  (S832A) | 13.9 | 37 | 5.5 | 10 | 19.9 | 13.4 | Outcome of paclitaxel and carboplatin therapy in ovarian cancer | OR=1.5 | (Hedditch et al. 2014) | 1,058 |
| ***TAP1*** | | | | | | | | | | |  |
| rs1135216 | Missense(D697G) | 14.6 | 23.4 | 15.9 | 21.8 | 18.6 | 15.8 | Increased risk of psoriasiform reactions by anti-TNFα drugs | OR=0.009 | (Cabaleiro et al. 2016) | 161 |
| ***ABCB4* (MDR3)** | | | | | | | | | | |  |
| rs1149222 | Intron | 80.0 | 39.5 | 59.8 | N.A. | 77.9 | 80.7 | Incerased risk of anthracycline-induced cardiotoxicity | OR=1.9 | (Visscher et al. 2012) | 156+188+96 |
| ***ABCB5*** | | | | | | | | | | |  |
| rs17143212 | Missense  (T131I) | 27.9 | 7.1 | 11.1 | 79.5 | 1.0 | 1.3 | Increased haloperidol-induced Toxicity | p= 0.034 | (Zheng et al. 2015) | 85 |
| ***ABCB11*** | | | | | | | | | | |  |
| rs2287622 | Missense  (V444A) | 59.7 | 56.6 | 72.6 | 57.5 | 42.2 | 55.9 | Increased rosuvastatin exposure | p=0.003 | (Soko et al. 2018) | 12 |

**Associated references:**

Cabaleiro T, Prieto-Pérez R, Navarro R, Solano G, Román M, Ochoa D, Abad-Santos F, Daudén E (2016) Paradoxical psoriasiform reactions to anti-TNFα drugs are associated with genetic polymorphisms in patients with psoriasis. The Pharmacogenomics Journal 16: 336-340. doi: 10.1038/tpj.2015.53

Hedditch EL, Gao B, Russell AJ, Lu Y, Emmanuel C, Beesley J, Johnatty SE, Chen X, Harnett P, George J, Group AOCS, Williams RT, Flemming C, Lambrechts D, Despierre E, Lambrechts S, Vergote I, Karlan B, Lester J, Orsulic S, Walsh C, Fasching P, Beckmann MW, Ekici AB, Hein A, Matsuo K, Hosono S, Nakanishi T, Yatabe Y, Pejovic T, Bean Y, Heitz F, Harter P, du Bois A, Schwaab I, Hogdall E, Kjaer SK, Jensen A, Hogdall C, Lundvall L, Engelholm SA, Brown B, Flanagan J, Metcalf MD, Siddiqui N, Sellers T, Fridley B, Cunningham J, Schildkraut J, Iversen E, Weber RP, Berchuck A, Goode E, Bowtell DD, Chenevix-Trench G, deFazio A, Norris MD, MacGregor S, Haber M, Henderson MJ (2014) ABCA transporter gene expression and poor outcome in epithelial ovarian cancer. Journal of the National Cancer Institute 106. doi: 10.1093/jnci/dju149

Soko ND, Chimusa E, Masimirembwa C, Dandara C (2018) An African-specific profile of pharmacogene variants for rosuvastatin plasma variability: limited role for SLCO1B1 c.521T&gt;C and ABCG2 c.421A&gt;C. The Pharmacogenomics Journal 78: 330. doi: 10.1038/s41397-018-0035-3

Tsai MY, Ordovas JM, Li N, Straka RJ, Hanson NQ, Arends VL, Arnett D (2010) Effect of fenofibrate therapy and ABCA1 polymorphisms on high-density lipoprotein subclasses in the Genetics of Lipid Lowering Drugs and Diet Network. Molecular genetics and metabolism 100: 118-122. doi: 10.1016/j.ymgme.2010.03.001

Visscher H, Ross CJD, Rassekh SR, Barhdadi A, Dubé M-P, Al-Saloos H, Sandor GS, Caron HN, van Dalen EC, Kremer LC, van der Pal HJ, Brown AMK, Rogers PC, Phillips MS, Rieder MJ, Carleton BC, Hayden MR, Consortium CPNfDS (2012) Pharmacogenomic Prediction of Anthracycline-Induced Cardiotoxicity in Children. Journal of Clinical Oncology 30: 1422-1428. doi: 10.1200/JCO.2010.34.3467

Voora D, Shah SH, Reed CR, Zhai J, Crosslin DR, Messer C, Salisbury BA, Ginsburg GS (2008) Pharmacogenetic Predictors of Statin-Mediated Low-Density Lipoprotein Cholesterol Reduction and Dose Response. Circulation: Cardiovascular Genetics 1: 100-106. doi: 10.1161/CIRCGENETICS.108.795013

Zheng M, Zhang H, Dill DL, Clark JD, Tu S, Yablonovitch AL, Tan MH, Zhang R, Rujescu D, Wu M, Tessarollo L, Vieira W, Gottesman MM, Deng S, Eberlin LS, Zare RN, Billard J-M, Gillet J-P, Li JB, Peltz G (2015) The Role of Abcb5 Alleles in Susceptibility to Haloperidol-Induced Toxicity in Mice and Humans. PLoS Medicine 12: e1001782. doi: 10.1371/journal.pmed.1001782
